# Society of the Alumni, Veterinary Department University of Pennsylvania

**Published:** 1901-08

**Authors:** E. M. Ranck

**Affiliations:** Secretary-Treasurer, 422 N. 41st St., Phila.


Secretary.

### SOCIETY OF THE ALUMNI, VETERINARY DEPARTMENT UNIVERSITY OF PENNSYLVANIA.

The annual banquet of the alumni of the Veterinary Department of the University of Pennsylvania was held at Philadelphia, June 12, 1901, at the

Rathskeller, Fifth and Ludlow Streets. Those present were Drs. S. J. J. Harger, James M. Mecray, H. D. Paxson, J. M. Carter, H. D. Martein, Charles W. Williams, Charles Lintz, T. J. Kean, W. H. Ridge, Edgar W. Powell, B. F. Senseman, M. E. Conard, F. W. Mackie, Charles R. Walters, Oscar M. Norton, Frederick Stehle, F. S. Carlisle, C. S. Shore, F. G. Woodward, J. W. Hayman, Samuel H. Gilliland, J. A. James, C. J. Marshall, James Beatty, and E. M. Ranck, of the alumni, and W. Horace Hoskins, of the American Veterinary College, New York.

The business meeting was called to order by President Marshall at 7.15 to consider the formation of a permanent alumni society of the Veterinary Department of the University of Pennsylvania. The President, having appointed a committee to prepare a constitution suitable for this purpose sometime previous, called for a report after some explanatory remarks. Some of the older alumni stated that there had been a regular organized society at one time, but no records were at hand nor could be obtained, so the members adopted the following constitution:

Constitution of the Society of the Alumni of the Veterinary Department of the University of Pennsylvania:

ARTICLE I.—*Name.*

The name of this Association shall be the Society of the Alumni of the Veterinary Department of the University of Pennsylvania.

ARTICLE II.—*Object.*

The object of the Society shall be to sustain and advance the interests and influence of the Veterinary Department of the University of Pennsylvania, to collect and preserve records of the graduates, to promote friendly relations between the alumni, and to extend the progress of veterinary science.

ARTICLE III.—*Members.*

SECTION 1. Any graduate of the University of Pennsylvania is eligible to membership upon payment of the annual dues.

SEC. 2. Honorary members may be elected at any stated meeting of the Society.

SEC. 3. Any member may become a life member upon the payment of twenty-five dollars ($25), which shall form part of a permanent fund.

ARTICLE IV.—*Officers.*

SECTION 1. The officers of the Society shall be a President, a Vice-President, a Secretary-Treasurer, an Historian and an Executive Committee.

SEC. 2. The President, or, in his absence, the Vice-President, shall preside at the meetings of the Society.

SEC. 3. The Secretary and Treasurer shall keep correct minutes of the proceedings, issue notices of all meetings, have charge of the correspondence of the Society, receive all moneys, make all payments ordered by the Executive Committee, keep correct accounts of all receipts and expenditures, and present a report at the annual meeting of the Society.

SEC. 4. The Historian shall collect all data which will interest the Society from an historical stand-point, and prepare a report for the regular meetings.

SEC. 5. The Executive Committee shall be composed of five members—the Vice-President, Secretary-Treasurer, and three members—who shall be elected at the annual meetings. It shall have control of all business of the Society, subject to the direction of the Society. It shall select members to fill such offices as may become vacant between the annual meetings. The Vice-President of the Society shall be chairman of this committee, and the Secretary-Treasurer of the Society shall be the Secretary. The proceedings of the meetings must be preserved and reported at the regular meetings of the Society.

ARTICLE V.—*Meetings.*

SECTION 1. The annual meetings shall take place in the City of Philadelphia on Commencement Day or upon such days as the Executive Committee shall select. There shall be but one meeting each year, unless otherwise ordered by a majority of the Executive Committee.

SEC. 2. Seven members shall constitute a quorum at any regular or special meeting.

ARTICLE VI.—*Election.*

Officers shall be elected by ballot at the annual meeting. The officers shall be a President, Vice-President, Secretary-Treasurer, an Historian, and three members of the Executive Committee.

ARTICLE VII.—*Dues.*

SECTION 1. The dues shall be at least one dollar a year.

SEC. 2. Members in arrears two years shall be suspended.

SEC. 3. The Executive Committee shall have power to reinstate the delinquents upon payment of arrearages to time of suspension.

ARTICLE VIII.—*Expulsion.*

Members may be expelled when such action is recommended in writing by the Executive Committee after a full investigation, if supported by two-thirds vote at any regular meeting.

ARTICLE IX.—*Order of Business.*

1. Reading of minutes.
2. Reports of officers and committees.
3. Election of members.
4. Collection of dues.
5. Nomination and election of officers.
6. Unfinished business.
7. New business.
8. Addresses.
9. Seating of officers.
10. Adjournment.

ARTICLE X.—*Committees.*

SECTION 1. There shall be a special committee of three appointed by the President each year to assist directly or indirectly in maintaining and promoting the undergraduate organization, the Veterinary Medical Association of the University of Pennsylvania.

Sec. 2. There shall be a special committee of three appointed by the President at each annual meeting to be known as the "Library Committee." It shall be the duty of this committee to assist in the formation of a library at the Veterinary Department of the University of Pennsylvania, for the use of the undergraduates and alumni, and for this purpose the committee may co-operate with the Faculty of the school and with the undergraduates upon such terms as seem favorable and subject to the approval of this Society.

Sec. 3. There shall be a committee of three, to be appointed by the President at each annual meeting, to collect all papers, communications, and essays written or published by magazines, periodicals, newspapers, etc., which relate to veterinary medicine, and give a report of their collections at the annual meetings, the manuscript collected to go to the library above provided for in Section 2.

### Article XI.—*Amendments.*

Amendments may be made to this Constitution at any of the annual meetings; provided, that due notice has been given to the members, and that at least two-thirds of the members present approve of its adoption.

After the adoption of the above Constitution the President called for the election of officers for the ensuing year, which resulted in the following: *President*, C. J. Marshall; *Vice-President*, W. H. Ridge; *Secretary-Treasurer*, E. M. Ranck; *Historian*, J. M. Carter. *Executive Committee*—Charles W. Williams, Leonard Pearson, S. J. J. Harger.

The Society then adjourned to the banquet table to welcome the graduates of the current year, C. J. Marshall officiating as toastmaster for the occasion.

The following members were called upon for addresses: S. J. J. Harger, W. H. Ridge, J. M. Carter, M. E. Conard, Charles Williams, H. D. Paxson, H. D. Martein, B. F. Senseman, T. J. Kean, W. Horace Hoskins, S. H. Gilliland, and other members present. The Committee on Arrangements must be complimented upon the faithful work performed under the leadership of C. J. Marshall, who is untiring in his efforts to promote good feeling and good fellowship among his professional brethren. Owing to the lateness of the hour the President deferred making his appointments on the various committees until some future time.

Thus a well-organized and complete Society has been launched which we hope will be a benefit to our alma mater and to the individual alumni as well as the community at large. The success of its future depends on all of the eligible graduates to enroll their names and help support and further its interests.

E. M. Ranck,
Secretary-Treasurer,
422 N. 41st St., Phila.

Note.—Will the general alumni of the University of Pennsylvania, Veterinary Department who read the above please forward their names and addresses to the Secretary-Treasurer, so they can be enrolled on the list. Date of graduation included. E. M. Ranck.

